# Public Expectations for Food and Drug Administration Approval of AI-Based Clinical Decision Support Tools: Quantitative Study

**DOI:** 10.2196/84315

**Published:** 2026-05-12

**Authors:** Gloria Maria Carmona Clavijo, Paige Nong, Sean Tan, Jodyn Platt

**Affiliations:** 1 TIERRA - Trust, Innovation & Ethics Research for Responsible AI Department of Learning Health Systems University of Michigan Ann Arbor, MI United States; 2 Division of Health Policy and Management School of Public Health University of Minnesota Minnesota, MN United States

**Keywords:** artificial intelligence, health policy, ethical and legal, learning health systems, public opinion

## Abstract

**Background:**

Regulation of artificial intelligence (AI) has been slow relative to the pace of its integration into health care. Several AI diagnostic tools for diabetic retinopathy (DR) have already received Food and Drug Administration (FDA) clearance, making it a timely and concrete example for exploring public perspectives on regulatory approval. The scope of FDA regulation of AI tools is being explored, and public attitudes about regulatory oversight should inform these discussions and are explored in this paper. Prior research suggests that comfort, trust, and political orientation shape views on government regulation and emerging technologies, potentially affecting support for oversight of AI in health care.

**Objective:**

This study assessed the perceived importance of FDA approval for AI-supported clinical decision support tools, with DR as the use case. We explored how comfort with AI tool developers, trust in data sharing, political affiliation, and demographic characteristics relate to the importance of FDA approval among US adults.

**Methods:**

A national survey was conducted in 2023 using the NORC AmeriSpeak Panel, a probability-based sample including 1787 respondents, with a subset of 982 participants answering questions about a use case describing an AI tool for identifying DR. Participants rated the importance of FDA approval for such tools on a 4-point Likert scale, with responses dichotomized between high and low perceived importance. Logistic regression models assessed associations between this outcome and predictors including comfort with AI tool developers, trust in data sharing, political affiliation, and demographic characteristics.

**Results:**

Among the 982 respondents presented with the DR use case, 658 (67%) indicated that FDA approval was “fairly” or “very” important. Statistically significant factors associated with the outcome (“It is important that the AI tool is approved by the FDA”) included higher comfort with using the tool (odds ratio [OR] 1.44, 95% CI 1.11-1.87; *P*=.006), comfort with developers from private companies (OR 1.38, 95% CI 1.09-1.76; *P*=.008), and hospitals (OR 1.60, 95% CI 1.25-2.05; *P*<.001). Trust in responsible data sharing (OR 1.25, 95% CI 1.05-1.5; *P*=.01) and higher education (OR 1.64, 95% CI 1.02-2.62; *P*=.04) also predicted higher support. Lean or strong Republicans (OR 0.43, 95%CI 0.3-0.6; *P*<.001) and Independents (OR 0.63, 95% CI 0.42-0.96; *P*=.03) were less likely to view FDA approval as important, as were Black (OR 0.50, 95% CI 0.34-0.77; *P*<.001) and Hispanic (OR 0.57, 95% CI 0.38-0.86; *P*=.007) respondents compared with White respondents.

**Conclusions:**

This study offers insights into public attitudes regarding FDA oversight of AI-based clinical decision support tools. Findings highlight how comfort, trust, and lower confidence from marginalized communities and some political groups shape perceived importance of FDA approval, offering a point for broader applications in health care AI governance. These factors should be better considered as health systems work to ensure trustworthy implementation of new AI technologies.

## Introduction

The integration of artificial intelligence (AI) into diagnostic workflows has gained attention due to its potential to improve efficiency and accuracy. While there is high variability in the quantity and quality of AI tools across medical specialties, diabetic retinopathy (DR)—a prevalent diabetes-related eye disease with significant public health impact—has seen notable progress [1-3]. AI tools can detect DR from retinal images, and several tools have already received Food and Drug Administration (FDA) clearance [4,5]. DR offers a well-known, real-world example of a regulated AI diagnostic tool, making it a timely use case for examining public attitudes toward this type of AI application.

AI systems used in diagnostic imaging vary in their level of autonomy [6]. Some function as AI-based clinical decision support (AI-CDS) tools, assisting clinicians in interpreting images, while others operate as autonomous diagnostic systems with the capacity of generating screening decisions without clinician interpretation [7]. Both types may be evaluated through FDA regulatory pathways for medical devices, with clearer oversight responsibilities for fully autonomous systems [8]. When an AI-CDS tool provides recommendations to clinicians, the FDA may still have a role in reviewing the tool, but the guidance is less clear. In this study, we focused on a use case of AI that supports diagnostic decision-making, in which the FDA may have a role. We then evaluated whether such oversight is important to patients. We were motivated by recent research on AI regulation that emphasizes the relationships between trustworthiness, risk acceptability, and public perceptions of oversight.

Studies show that regulatory frameworks influence societal acceptance of AI [9], that support for governance is shaped by perceived risk and developer credibility [10], and that AI literacy and anxiety affect how individuals evaluate the necessity of oversight [11]. As diagnostic applications of AI expand, little is known about public perceptions and expectations about their oversight, including FDA approval [12,13]. Attitudes about the FDA and regulation may be shaped by whether there is a perceived need. If there is confidence in local control and trust in health systems, sufficient comfort with an AI tool and its developers, and a lack of confidence in government, regulatory oversight may be perceived as less important. Theories of trust in health would predict that greater comfort and confidence on the part of a truster (in this case, patients) would diminish the need for regulation and formal governance practices over the trustee (in this case, the AI tool) [14]. With greater comfort and confidence in an AI tool, patients may not need additional oversight to ensure safety and quality.

**Figure 1:**
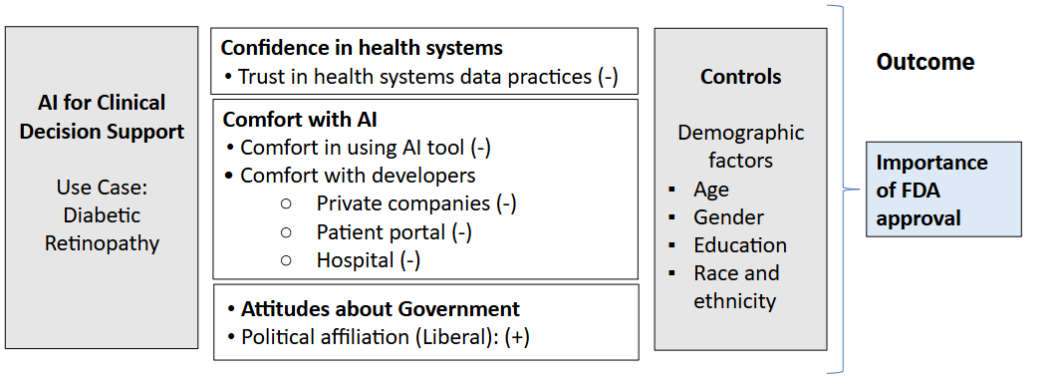
Predictors and hypothesized relationship to importance of Food and Drug Administration approval for artificial intelligence for clinical decision support. AI: artificial intelligence; FDA: Food and Drug Administration.

More specifically, trustworthiness of health systems is likely to have an impact on the importance of FDA approval; we hypothesize that if health systems are adequately vetting AI tools, the perceived need for FDA approval may be diminished. Public attitudes about the importance of FDA approval may also be shaped by their personal feelings about AI. For example, someone who is comfortable with AI tools [15] may view FDA approval as less essential [16], while those who do not see the health system as having a good track record acting on its own may see regulatory oversight as more important [17]. Similarly, AI tools that are developed by trustworthy parties may also diminish the importance of FDA approval. Comfort and confidence in the capabilities of AI tools, developers, and health systems are each personal attitudinal attributes of trust that shape how individuals engage with new technologies and institutions [18-20] and may be associated with public perceptions on whether formal regulatory oversight is necessary. At the same time, and as public trust in health and research has declined, the politicization of science has increased [21,22]. Political affiliation, as a personal attribute, is likely to play a role in patient perspectives on the importance of regulatory oversight. Individuals identifying as more conservative or Republican often express greater skepticism toward government intervention, centralized data systems, and emerging technologies than Democrats. These attitudes may extend to issues such as whether FDA approval is perceived as necessary in the context of health care AI. The conceptual model in Figure 1 shows these factors as we hypothesize, they will be related to perceived need for FDA approval of the DR tool positively or negatively.

In this study, we focus on FDA clearance as a distinct and standardized regulatory measure. Unlike more abstract notions of regulation, oversight, or certification, FDA clearance is a formal federal evaluation of safety and effectiveness for a specific diagnostic tool [23]. Through the 510(k) process, the FDA issues clearance letters only after reviewing device-specific evidence, providing a concrete and publicly official stamp of approval [8,23]. Distinguishing this type of FDA approval from generic regulatory concepts allows us to explore how individuals respond to a clearly defined and institutionally established form of oversight.

While prior studies have explored general attitudes toward AI oversight, this study extends prior work by assessing the perceived importance of FDA approval for a diagnostic support AI tool (DR), and to identify factors associated with this perception, including comfort with the specific AI tool, the AI developers, and health system’s track record (trust in data sharing), political identity, and demographic factors among adults living in the United States (Figure 1). By focusing on a concrete AI application, we provide a more integrated understanding of influences shaping public attitudes toward real-world regulatory oversight of diagnostic AI tools.

## Methods

### Study Design

In 2023, we conducted a survey using the National Opinion Research Center’s AmeriSpeak Panel, a probability-based sample of US residents, with an oversampling of Black or African American and Hispanic or Latino respondents. The total sample size was 1787 participants, with 982 participants responding to a hypothetical use case related to the use of AI tools supporting the diagnosis of DR:

You were recently diagnosed with diabetes. As a part of your treatment, you are routinely screened for diabetic retinopathy, a complication of diabetes that affects the eyes and can lead to blindness. This kind of screening involves taking an image of the eye to look for damaged blood vessels. Your doctor uses an AI tool that analyzes your medical images to identify diabetic retinopathy. For you, how true are the following statements?

### Measures

The outcome of interest was the perceived importance of FDA approval of the AI tool in the use case. We asked participants how true the following statement was for them: “It is important to me that the FDA has approved this AI tool” on a scale from not at all true (score=1) to very true (score=4). We generated a binary dependent variable “Importance of FDA approval,” grouping “not at all” or “somewhat true” into one category, and “fairly true” or “very true” responses into another, based on the distribution of the variable.

### Statistical Analysis

We then examined factors that might predict the perceived importance of FDA approval.

Specifically, respondents rated their agreement (1=not at all true to 4=very true) with statements capturing (1) comfort with the AI tool (“I am comfortable with this AI tool being used in this way”), (2) comfort with private company developers (“I would be comfortable with private companies making this AI tools such as IBM, Google, Johnson & Johnson”), (3) comfort with hospitals developing the tool (“I am comfortable with my hospital making this AI tool”), (4) comfort with patient portal companies developing the tool (“I would be comfortable with the company that makes my patient portal making this AI tool”), and (5) trust in responsible data sharing (“The organization that have my health information and share it have a good track record of using it responsibly”). These Likert scale predictors were treated as continuous variables, consistent with prior social science and health research. Treating these variables as continuous allows us to see how changes across the response levels are related to the outcome [24,25].

Political orientation was assessed as “strong/not so strong or lean Democrat,” “Do not lean,” and “strong/not so strong or lean Republican.” Indicator variables were created to evaluate categorical and ordinal variables (political orientation, sex, age, race or ethnicity, and education).

We conducted univariable and multivariable logistic regression models to identify predictors in the perceived importance of FDA approval for AI diagnostic tools using questions evaluating comfort with the AI tool and AI developers, and trust in health data sharing, political identity, and demographic factors. As a sensitivity analysis, we re-estimated models, treating Likert variable predictors as categorical variables and conducted ordinal logistic regression modeling the 4-level outcome variable. To assess the overall contribution of the categorical variables to the model, multicollinearity among predictors was assessed using variance inflation factors (VIFs). Sensitivity analyses are provided in Multimedia Appendix 1.

### Ethical Considerations

This study was considered exempt by the University of Michigan Institutional Review Board. Participants were compensated for their time according to standard National Opinion Research Center remuneration policies, US $4 per survey. The National Opinion Research Center obtained participants’ written informed consent. To ensure respondents privacy and confidentiality, identifying information was removed. As a result, the dataset used in the study were anonymized, and not personal identifiable information was accessible to the research team.

## Results

### Participant Characteristics

Among 982 respondents, 537 (54.7%) were female and 445 (45.4%) were male; 270 (27.8%) were Black, 237 (24.1%) were Hispanic, 429 (43.4%) were White, and 46 (4.7%) were other race groups (non-Hispanic).

The mean of comfort with the use of the AI tool was 2.56 (SD 0.95). Participants expressed moderate comfort with the DR AI tool being developed by hospitals (mean 2.45, SD 0.96) and private companies such as IBM, Google, and Johnson & Johnson (mean 2.34, SD 0.94), while the lowest mean values were reported for comfort with patient portal companies developing such tools (mean 2.32, SD 0.94). Participants also showed moderate agreement that organizations responsible for health data sharing have a good track record of using information responsibly (mean 2.37, SD 0.92). A majority of participants identified as strong or lean Democratic (541/982, 55.1%).

### Perceived Importance of FDA Approval

Overall, of the 982 participants, 658 (67%) stated that it was “fairly true” or “very true” that FDA approval of the AI tool was important (Table 1).

**Table 1:** Descriptive statistics of variables used in logistic regression: demographic factors, and independent and dependent variables used in the theoretical framework (n=982).

Characteristics		Value
**Sex, n (%)**		
	Male	445 (45.3)
	Female	537 (54.7)
**Age group (y), n (%)**		
	18-29	157 (16)
	30-44	281 (28.6)
	45-59	222 (22.7)
	>60	322 (32.8)
**Race or ethnicity, n (%)**		
	Black American, non-Hispanic	270 (27.8)
	Hispanic	237 (24.1)
	Other, non-Hispanic	46 (4.7)
	White, non-Hispanic	429 (43.4)
**Education, n (%)**		
	Less than or high school graduate	237 (24.1)
	Some college or associate degree	407 (41.5)
	Bachelor’s degree	194 (19.8)
	Postgraduate study or professional degree	144 (14.7)
**Independent variables: attributes, mean (SD)**		
	“I am comfortable with this AI^a^ tool being used in this way.”^b^	2.56 (0.95)
	“I would be comfortable with a private company (such as IBM, Google, and Johnson & Jonhson) making this AI tool.”^b^	2.34 (0.94)
	“I am comfortable with my hospital making this AI tool.”^b^	2.45 (0.96)
	“I would be comfortable with the company that makes my patient portal making this AI tool.”^b^	2.32 (0.94)
	“The organizations that have my health information and share it, have a good track record of using it responsibly.”^b^	2.37 (0.92)
**Political affiliation, n (%)**		
	Strong or not so strong or lean Democrat	541 (55.1)
	Do not lean	164 (16.7)
	Strong or not so strong or lean Republican	277 (28.2)
**Dependent variable: outcome, mean (SD)**		
	Importance of FDA^c^ in approving AI tools used for diagnosis^d^	0.67 (0.47)

^a^AI: artificial intelligence.

^b^4-point scale: 1=not at all true; 4=very true.

^c^FDA: Food and Drug Administration.

^d^Binary variables coded as follows: 0=not true (n=122, 12.4%) or somewhat true (n=205, 20.9%); 1=fairly true (n=232, 23.6%) or very true (n=433, 43.1%).

### Predictors of FDA Approval Importance

In multivariable logistic regression analysis, higher perceived importance of FDA approval was associated with greater comfort with the DR AI tool being used (odds ratio [OR] 1.44, 95% CI 1.11-1.87; *P*=.006), comfort with the tool being developed by private companies (OR 1.38, 95% CI 1.09-1.76; *P*=.008), and hospitals (OR 1.60, 95% CI 1.25-2.05; *P*<.001). Trust in responsible data sharing was also positively associated (OR 1.25, 95% CI 1.05-1.5; *P*=.01) with the outcome. Political affiliation played a significant role: lean-strong Republicans (OR 0.43, 95% CI 0.3-0.6] *P*<.001) and Independents (OR 0.63, 95% CI 0.42-0.96; *P*=.03) demonstrated lower odds of considering FDA approval important. Black American (OR 0.50, 95% CI 0.34-0.77; *P*<.001) and Hispanic (OR 0.57, 95% CI 0.38-0.86 *P*=.007) participants also showed lower odds of considering FDA approval important. Meanwhile, those with higher levels of education were more likely to consider FDA approval of AI diagnostic tools important (OR 1.64, 95% CI 1.02-2.62; *P*=.04; Table 2). Sensitivity analyses demonstrated substantively similar findings. Multimedia Appendix 1 shows the results of the regression model in which we treat all independent variables as categorical. Additionally, all VIF values were <3 (mean VIF 1.57, SD 0.66), indicating low multicollinearity and that predictors were empirically distinguishable.

**Table 2:** Logistic regression analysis of the importance of the Food and Drug Administration in approving artificial intelligence tools used for the diagnosis of diabetic retinopathy (n=982).

Predictors		Univariable			Multivariable	
		OR^a^	*P* value	OR		*P* value
**Personal comfort**						
	“I am comfortable with this AI tool being used in this way.”^b^	2.41	*<.001* ^c^	1.44		*.006*
**Private companies**						
	“I would be comfortable with private companies making this AI tool such as IBM, Google, and Johnson & Johnson.”^b^	2.21	*<.001*	1.38		*.008*
**Patient portal**						
	“I would be comfortable with the company that makes my patient portal making this AI tool.”	1.85	*<.001*	0.93		.56
**Comfort with the hospital**						
	“I am comfortable with my hospital making this AI tool.”^b^	2.27	*<.001*	1.60		*<.001*
**Trusted health data sharing**						
	“The organizations that have my health information and share it, have a good track record of using it responsibly.”	1.45	*<.001*	1.25		*.01*
**Political affiliation**						
	Lean, not so strong, and strong Democrat (reference)	—^d^	—	—		—
	Do not lean—Independent	0.44	*<.001*	0.63		*.03*
	Lean, not so strong, and strong Republican	0.50	*<.001*	0.43		*<.001*
**Sex**						
	Male (reference)	—	—	—		—
	Female	0.10	.98	1.19		.26
**Age (y)**						
	18-29 (reference)	—	—	—		—
	30-44	0.87	.51	0.74		.20
	45-59	0.94	.76	0.74		.23
	≥60	1.88	*.003*	1.36		.21
**Race or ethnicity**						
	Black American, non-Hispanic	0.61	*.003*	0.50		*<.001*
	Hispanic	0.58	*.001*	0.57		*.007*
	Other	0.52	.04	0.54		.10
	White, non-Hispanic (reference)	—	—	—		—
**Education**						
	Less than or high school (reference)	—	—	—		—
	Some college or associate degree	1.63	*.004*	1.45		.06
	Bachelor’s degree	2.08	*<.001*	1.63		*.04*
	Postgraduate study or professional degree	2.18	*.001*	1.35		.27

^a^OR: odds ratio.

^b^4-point scale: 1=not at all true; 4=very true.

^c^Italicized indicate *P*<.05.

^d^Not applicable.

## Discussion

### Principal Findings

Overall, we find that the public is supportive of FDA regulation of AI tools, and that need for regulation is not supplanted by comfort with or trust in AI, its developers, or health systems. Our findings suggest that people who are comfortable with the use of the AI tool for DR are more likely to desire FDA approval of tool, which may also suggest that people, even the most comfortable AI users, may be using the FDA approval process as a proxy for trust in the AI-CDS tool’s safety and effectiveness. This is surprising given our hypothesis that greater comfort might diminish the need for additional oversight. These findings are consistent with public expectations of having more rather than less regulation when it comes to AI and the companies that build them [12,18] and suggest that even with greater familiarity with AI tools, regulation is still a critical component of overall AI governance. This is likely to be true regardless of who develops the tool, though Pew Research shows that Americans favor more regulation of Big Tech companies (51% in 2024) [16,26]. Greater attention and effort must be given to ensure that regulatory agencies have broad and clear authority to regulate AI tools.

Additionally, protecting the legitimacy of the FDA and other regulatory agencies may also be key to public trust in AI tools. Transparency is a key FDA guiding principle [27].

Our findings suggest that hospitals and AI developers may need to seek AI tools that are approved or have gone through a reliable certification process to indicate their safety and effectiveness. Political affiliation was a statistically significant predictor of the perceived importance of FDA regulation—lean or strong republicans (OR 0.43, 95% CI 0.3-0.6; *P*<.001) and Independents (OR 0.63, 95% CI 0.42-0.96; *P*=.03) were less likely to rate it as important- highlighting the potential influence of political considerations on perception of FDA regulation.

The statistical significance of political affiliation, Lean or strong Republicans (OR 0.43, 95% CI 0.3-0.6; *P*<.001) and Independents (OR 0.63, 95% CI 0.42-0.96; *P*=.03), as a predictor of the importance of FDA regulation highlights the FDA’s inability to avoid political considerations [28].

### Implications for Research and Practice

Our findings also underscore the need to deepen our understanding of how public trust in AI tools is shaped. Rather than assuming transparency is both necessary and sufficient for trust, future research may consider whether trust may drive demand for transparency and oversight. Approval or certification processes from trusted entities, such as the FDA, could serve to signal the legitimacy of and increase public comfort with AI tools in health care. These insights point to the opportunity to further develop a conceptual framework that connects comfort with AI, perceived safety, regulatory presence, and public demand for accountability. Ultimately, identifying where that threshold of comfort lies could inform more effective strategies for regulatory engagement and public communication.

### Limitations

Our findings are based on DR as a use case and may not fully generalize attitudes toward FDA oversight of other AI tools, particularly new or emerging tools. Further research should explore additional clinical use cases to assess the broader applicability of these findings. Additionally, our analysis was focused on identifying associations rather than establishing causality, highlighting the need for future qualitative and longitudinal research. This is especially important considering the rapid evolution of AI health tools.

### Conclusions

These findings suggest that the public may perceive FDA approval of AI diagnostic tools as important but also highlights the role of individual comfort with AI, trust in organizations, political views, age, and education level may influence these perceptions. As AI-CDS tools continue to evolve, understanding these perceptions can help guide the development of responsible and trustworthy health systems. These results are based on a nationally representative sample of US adults and may not generalize to other populations or health care contexts. Further research could explore equity implications and the experiences of diverse groups.

## Data Availability

The datasets generated or analyzed during this study are available from the corresponding authors on reasonable requests.

## Appendix

Multimedia Appendix 1 Sensitivity analysis of the importance of the Food and Drug Administration in approving artificial intelligence tools used for diagnosis of diabetic retinopathy (n=982).

## Abbreviations

AI: artificial intelligence
AI-CDS: AI-based clinical decision support
DR: diabetic retinopathy
FDA: Food and Drug Administration
OR: odds ratio
VIF: variance inflation factor
