# Supplementary material for: Public Expectations for Food and Drug Administration Approval of AI-Based Clinical Decision Support Tools: Quantitative Study
Source: JMIR AI. 2026 May 12;5:e84315. doi: 10.2196/84315 (PMC13213324; doi:10.2196/84315)
Supplement: Multimedia Appendix 1 [file ai_v5i1e84315_app1.docx]

**Table 1.** Sensitivity analysis of the importance of Food and Drug Administration in approving artificial intelligence tools used for diagnosis of diabetic retinopathy (n=982).

|  |  | Multivariable | |
| --- | --- | --- | --- |
| Predictors | Description | OR | P value |
| Personal comfort | I am comfortable with this AI tool being used in this way. |  |  |
|  | Not true (Ref) |  |  |
|  | Somewhat true | 1.52 | 0.18 |
|  | Fairly true | 2.81 | 0.003 |
|  | Very true | 2.60 | 0.027 |
| Private companies | I would be comfortable with private companies making this AI tool such as IBM, Google, Johnson & Johnson |  |  |
|  | Not true (Ref) |  |  |
|  | Somewhat true | 1.20 | 0.458 |
|  | Fairly true | 2.02 | 0.016 |
|  | Very true | 2.12 | 0.083 |
| Patient portal | I would be comfortable with the company that makes my patient portal making this AI tool |  |  |
|  | Not true (Ref) |  |  |
|  | Somewhat true | .839 | 0.504 |
|  | Fairly true | .831 | 0.546 |
|  | Very true | .83 | 0.682 |
| Comfort with | I am comfortable with my hospital making this AI tool |  |  |
| hospital | Not true (Ref) |  |  |
|  | Somewhat true | 1.14 | 0.644 |
|  | Fairly true | 2.80 | 0.001 |
|  | Very true | 2.82 | 0.015 |
| Trusted Health data sharing | The organizations that have my health information and share it have a good track record of using it responsibly |  |  |
|  | Not true (Ref) |  |  |
|  | Somewhat true | 1.37 | 0.159 |
|  | Fairly true | 1.83 | 0.021 |
|  | Very true | 1.78 | 0.052 |
| Political affiliation | Lean, not so strong, and Strong Democrat (Ref) |  |  |
|  | Do not lean – Independent | 0.44 | <.001 |
|  | Lean, not so strong, and Strong Republican | 0.50 | <.001 |
| Sex | Male (Reference) |  |  |
|  | Female | 0.10 | .980 |
| Age | 18-29 (Reference) |  |  |
|  | 30-44 | 0.87 | .510 |
|  | 45-59 | 0.94 | .761 |
|  | 60+ | 1.88 | .003 |
| Race/ ethnicity | White, non-Hispanic (Reference) |  |  |
|  | Black Americans, non-Hispanic | 0.61 | .003 |
|  | Other | 0.52 | .040 |
|  | Hispanics | 0.58 | .001 |
| Education | Less than or high school (Reference) |  |  |
|  | Some college / Associate degree | 1.63 | .004 |
|  | Bachelor's degree | 2.08 | <.001 |
|  | Post graduate study/Professional degree | 2.18 | .001 |
| OR: Odds Ratio. | |  | |
